# Factors Associated with ICU Mortality at Hawassa University Comprehensive Specialized Hospital (HUCSH)

**DOI:** 10.4314/ejhs.v32i3.5

**Published:** 2022-05

**Authors:** Abdi Bati Wotiye, Emnet Tesfaye Shimber, Biniyam A Ayele

**Affiliations:** 1 Internist, Assistant Professor of Gastroenterology and Hepatology All Africa Leprosy, Tuberculosis and Rehabilitation Training Centre (ALERT Center), Addis Ababa, Ethiopia. Po Box:5164; 2 Assistant Professor of Emergency and Critical Care Medicine, College of medicine and health sciences, Hawassa University, Ethiopia; 3 Assistant Professor of Neurology, Department of Neurology College of Health Sciences, Addis Ababa University, Addis Ababa, Ethiopia

**Keywords:** Critical care unit, mortality, respiratory support, acute kidney injury, aspiration pneumonia

## Abstract

**Background:**

Providing acute care to critically ill patients in intensive care unit (ICU) is a global necessity, regardless of health system capacity. The objectives of the present study were to assess the reasons for admission and clinical outcomes of adult patients admitted to general ICU at Hawassa University Comprehensive Specialized Hospital (HUCSH).

**Methods:**

A retrospective study based on a record review of logbook and charts of 310 adult patients admitted to general ICU of HUCSH between April 2012 and April 2017. Both descriptive analysis and inferential statistics were used.

**Results:**

The average age was 41 ± 17.9 years (range: 18–100 years). Males accounted 51.6%. The average duration of stay in the ICU was 5.3 ± 6.7 days (range 1–49 days). Cardiovascular disorders were the commonest cause of admission, accounted for 22.9%, followed by neurological disorders (17.7%), and trauma related illnesses (13.9%). Among 310 adults admitted during the study period, 23.1% required mechanical ventilator support; and 5.3% developed Hospital acquired infection. In-patient mortality rate was 45.8%. In multivariate analysis, the presences of aspiration pneumonia, and need for ventilator support, acute kidney injury, hospital acquired infection, and short ICU stays were associated with ICU mortality.

**Conclusion:**

The present study indicates high prevalence of ICU mortality among adults admitted to HUCSH during the study period. ICU mortality was associated with the presences of renal failure, hospital acquired infections, aspiration pneumonia and the need for mechanical ventilator

## Introduction

Intensive Care Unit (ICU) is a special unit of a hospital for patients with severe and life-threatening conditions requiring close intensive monitoring, specialized support using varies sophisticated equipment's and medications in order to maintain normal human physiologic function (1). ICUs in most of the developed world are high-technology facilities with the most advanced medical technology and skilled personnel. Caring for the critically ill patients is a challenge in developing countries, where health needs often outstrip available resources (2, 3). Globally, non-communicable diseases are the leading cause of intensive care unit Death, and the burden of disease are rising fastest among lower-income countries. The burden of cardiovascular diseases increased greatly in Ethiopia from 18% to 46% of all medical intensive care unit admissions over the past 30 years (4).

In a study done in Ethiopia, ICU mortality rate was 50.4%, with cardiovascular illness being the commonest cause of admission and death (5). Comparable results were reported from Tanzania and Nigeria, where ICU mortality rate was 41.4% (6) and 34.6% (7) respectively. There is a need to better define the epidemiology of sepsis in intensive care units (ICUs) around the globe. Sepsis remains a major health problem in ICU patients worldwide and is associated with high mortality rates. There is wide variation in sepsis rates, causative microorganisms, and outcome in ICU patients around the world. A history of liver cirrhosis or metastatic cancer, use of mechanical ventilation or renal replacement therapy, and Acinetobacter infection were independently associated with an increased risk of in-hospital death (8).

The neglect of systems to critical care in Africa is evident in the limited availability of intensive care units all across the continent. Moreover, there is great paucity of published works in area of intensive care units especially in Ethiopia, indicating wide gap to fill. Ethiopia, the second populous country in Africa, home to more than 110 million people have only hand full of university referral hospitals and ICU set up, which are often poorly equipped with necessary equipment's and well-trained health personnel. The aim of this study was to determine reasons for admission and outcome of patients admitted to general intensive care unit at Hawassa University comprehensive specialized hospital, the biggest hospital in Southern Ethiopia, built to serve more than 8 million people. We hope our effort to understand the clinical characteristics and outcome of patients admitted to our ICU will help to improve quality of care and clinical outcome of adult patients admitted to our ICU.

## Methods and Materials

**Study aim, area and setting**: In an effort to improve quality of critical care service currently delivered in Ethiopia, especially in southern part of Ethiopia, we aimed to understand pattern of disease, causes of ICU admission, and ICU mortality rate at Hawassa university comprehensive specialized hospital, so that the findings will guide the policy makers and future interventions in the region. The hospital was established in 1998 and it is the biggest hospital in the southern Ethiopia. The hospital has a capacity of 250 beds and has one open system general ICU with a capacity of 8 beds for comprehensive care of all critically ill patients from all specialties.

**Study design and period**: A retrospective study was conducted based on the ICU logbook and charts of patients admitted from April 2012 to April 2017.A total of 1554 patients were admitted during the study period. All adult patients aged 18 and above admitted to the ICU were included in the study. Patients with lost or incomplete data and patients who were dead on arrival were excluded from this review.

**Sample size calculation**: Since the reported prevalence of ICU mortality in Ethiopia has wide variation, we opted for 50% proportional (P) value for the present study. We calculated the sample size considering the following values: outcome proportion of 50%, margin of error of 5%, and confidence interval (CI) of 95% for finite population. Accordingly, we final sample size was 370. We assumed 5% attrition rate and the final sample size was N=380. Total of 70 medical records were excluded because of incompleteness and pediatrics admissions. Finally, a total of 310 patients were included in the final analysis.

**Study variables and Data collection process**: The dependent variable was clinical outcome. The independent variables were; Sociodemographic variables, diagnosis at admission, presence of comorbid illness, source, frequency and category of admission, vital signs at admission, interventions and Length of ICU stay. The dependent and independent variables were collected through checklist that was adapted by reviewing different literatures.

**Ethical consideration**: Ethical clearance was obtained from Hawassa University, School of medicine Institutional Review Board (IRB) with (Protocol number: IRB/040/09). Accordingly, letter of support was obtained from department of Internal medicine and chief clinical director of the hospital for Permission to conduct the study and access patient charts from the Archive. Confidentiality of the patients' charts were kept private, in which patients' address and other identifications (Card No) were removed before analysis.

**Statistical analysis**: Demographic data and clinical characteristics were first described by their range, means, and standard deviation. Association between outcome variable and different clinical characteristics were done using chi-square and crude odds ratio (OR) with 95% CI. The strength of association was presented using adjusted odds ratio with 95% CI and P-value < 0.05 was considered as statistically significant.

## Results

**Baseline characteristics of the study participants**: In the present review, total of 310 medical records of adults admitted to the HUCSH intensive care unit between 2012 and 2017 were included in the analysis. The median age was 36.5 (IQR 25.0 – 52.5) years. Males accounted 51.6% of admitted patients. The median duration of ICU stay was 3.0 (IQR 1.0 – 6.0) days. Out of the total 310 patients, 45.8% of the patients died in ICU. Lower level of consciousness on admission (GCS<9) was observed in 17.7% of the patients. Nearly one third of the patients were in some type of shock. Out of 310 patients, 23.5% (n=73/310) required mechanical ventilator support. Furthermore, 42% (n=77/176) developed acute kidney injury (AKI) while in ICU. Hospital acquired infection was reported in 5.3% (n=16/301) of the study participants. Of the total 310 patients, 23.9% developed aspiration pneumonia. In more than half (53.5%) of the patients, respiratory failure was reported as the immediate cause of death (Table 1).

**Table 1:** Baseline characteristics of the study participants (n=310)

Variables	Value
Age in years (median, IQR)	36.5 (25.0 – 52.5)
Male (n, %)	160 (51.6)
Duration of ICU stay in days (median, IQR)	3.0 (1.0 – 6.0)
Clinical outcomes (n, %)	
Improved	168 (54.2)
Death	142 (45.8)
Level of consciousness on ICU admission (n, %)	
GCS 15	163 (52.6)
GCS 13–14	28 (9)
GCS 9–12	64 (20.6)
GCS <9	55 (17.7)
Types of shock on ICU admission (n, %)	
No shock	241 (77.7)
Septic	21 (6.8)
Hypovolemic	12 (3.9)
Cardiogenic	36 (11.6)
Need for mechanical ventilation (n, %)	73 (23.5)
Acute kidney injury (n=176, %)	77 (42.0)
Hospital acquired infection (n=301, %)	16 (5.3)
Aspiration pneumonia (n, %)	74 (23.9)
Immediate causes of death (n=155, %)	
Respiratory failure	83 (53.5)
Multiorgan failure	31 (20.0)
Increased ICP	8 (5.2)
Cardiac arrest	11 (7.1)
Cardiorespiratory failure	7 (4.5)
Refractory shock	15 (9.7)

¶The denominator for all the variables is N=310, unless otherwise specified

**Figure 1:**
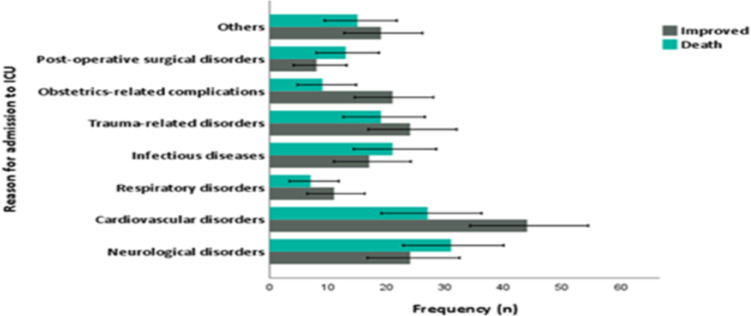
Bar graph showing the commonest cause of ICU admission along with respective case fatality rate.

The commonest reason for ICU admission was cardiovascular disorders, followed by neurological disorders (Figure 1). In the present review, neurological disorders, especially strokes were associated with high case fatality. In addition, higher case fatalities were observed in those individuals admitted to the ICU due to infectious diseases and post-operative surgical patients (Figure 1).

**Figure 2:**
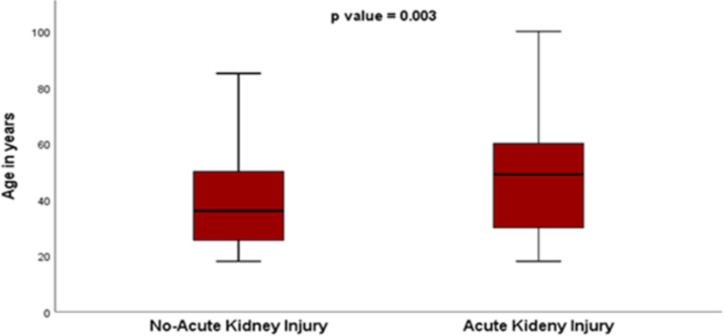
Box and whisker graph showing association between advanced age and occurrence of acute kidney injury (AKI)

**Factors associated with ICU mortality in study participants**: In the present study, no difference was observed between gender and ICU mortality. Similarly, no difference was seen in ICU mortality of those below and above age 36. Higher proportion of death was registered for those patients with duration of ICU stay below 3 days compared to those whom stayed more than 3 days in ICU (31.9% vs. 21.6% p<0.0001). Furthermore, those patients admitted to ICU with GCS below 13 died more compared to those with GCS above 13 (23.25 vs. 15.2% p<0.0001). No difference in ICU mortality was regarding presences of shock at the time of admission and presence of hospital acquired infection (Table 2). Higher proportion of ICU death was reported for admitted patients who requires mechanical ventilator support (p<0.0001), patients with acute kidney injury (p<0.0001), and aspiration pneumonia (p<0.0001) (Table 2). The proportion of AKI was significantly higher among older study participants compared to those who are young (p=0.003) (Figure 2).

**Table 2:** Risk factors associated with ICU mortality among study participants

Variables	Clinical outcomes		P-value
		
	Improved N=168 (54.2%)	Died N=142 (45.8%)	
Male	90 (29)	70 (22.3)	0.45
Female	78 (25.2)	72 (23.2)	
Age category			
≤ 36 years	88 (28.4)	67 (21.6)	0.36
> 36 years	80 (25.8)	75 (24.2)	
Duration of ICU stay			
≤ 3 days	67 (21.6)	99 (31.9)	<0.0001
> 3 days	101 (32.6)	43 (13.9)	
Level of consciousness			
GCS ≥ 13	121 (39)	70 (22.6)	<0.0001
GCS < 13	47 (15.2)	72 (23.2)	
Presence of shock on			
admission			
Yes	33 (10.6)	36 (11.6)	0.22
No	135 (43.5)	106 (34.2)	
Need for mechanical			
ventilation			
Yes	14 (4.5)	59 (19)	<0.0001
No	154 (49.7)	83 (26.8)	
Acute kidney injury			
Yes	28 (15.9)	49 (27.8)	<0.0001
No	74 (42)	25 (14.2)	
Hospital acquired infection			
Yes	6 (2)	10 (3.3)	0.20
No	158 (52.5)	127 (42.2)	
Aspiration pneumonia			
Yes	16 (5.2)	58 (18.7)	<0.0001
No	152 (49)	84 (27.1)	

**Figure 3:**
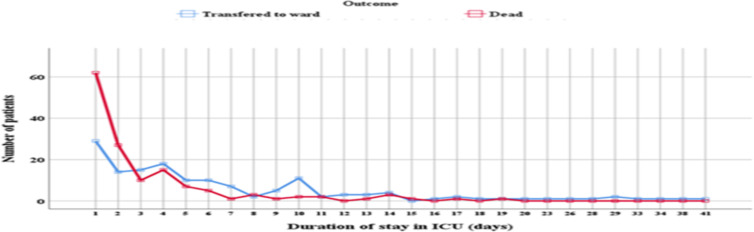
Line graph showing the relationship between shorter duration of admission and frequency of ICU mortality

**Logistic regression analysis of ICU mortality and covariates**: Shorter (<3 days) duration of ICU stay was associated with ICU mortality in both univariate (COR 3.47 95% CI 2.16 – 5.57 p<0.0001) and multivariate (AOR 4.44 95% CI 1.91 – 10.1 p<0.0001) logistic regression analysis (Table 3). In the present review, higher proportion of deaths were reported within the first two days of patient's ICU admission compared to those who were transferred to ward after clinical improvement (Figure 3). In multivariate analysis, ICU mortality was associated with the presence of the need for mechanical ventilator (AOR 0.30 95% CI 0.11 – 0.83 p=0.02); with the presence of acute kidney injury (AOR 0.22 95% CI 0.10 – 0.48, P<0.0001); hospital acquired infection (AOR 0.10 95% CI 0.02 – 0.53, P=0.007); and presence of aspiration pneumonia (AOR 0.10 95% CI 0.03– 0.36 p=0.001) when adjusted for the respective covariates (Table 3).

**Table 3:** Logistic regression analysis of ICU mortality and covariates

Covariates	Crude Odd Ratio (COR)			Adjusted Odd Ratio (AOR)		
	
	COR	95% CI	P value	AOR	95% CI	P value
Duration of ICU stay						
> 3 days	Ref.					
≤ 3 days	3.47	2.16 – 5.57	<0.0001	4.44	1.91 – 10.10	<0.0001
Level of consciousness						
GCS ≥ 13	Ref.					
GCS < 13	0.38	0.24 – 0.61	<0.0001	1.35	0.44 – 4.16	0.6
Shock on admission						
No	Ref.					
Yes	0.72	0.42 – 1.23	0.23	0.43	0.17 – 1.11	0.08
Ventilator support						
No	Ref.					
Yes	0.13	0.07 – 0.24	<0.0001	0.30	0.11 – 0.83	0.02
Acute kidney injury						
No	Ref.					
Yes	0.19	0.10 – 0.37	<0.0001	0.22	0.10 – 0.48	<0.0001
Hospital acquired infection						
No	Ref.					
Yes	0.48	0.17 – 1.36	0.17	0.10	0.02 – 0.53	0.007
Aspiration pneumonia						
No	Ref.					
Yes	0.15	0.08 – 0.28	<0.0001	0.10	0.03 – 0.36	0.001

## Discussion

In the present review, the median age was in the third decades with proportional male to female ratio and median ICU admission was three days. These results were consistent with prior reports from other referral hospitals in Ethiopia (1,2). The overall mortality rate of the ICU was 45.8 %. This is in congruent with studies reported from Ethiopia and Nigeria (2–6).

Cardiovascular disorders are the commonest reasons for ICU admission, followed by neurological disorders. Those patients admitted to the ICU with stroke had the highest case fatality. In this study, ICU deaths were significantly associated with shorter ICU stay and the need for mechanical ventilator, and the presence of aspiration pneumonia, acute kidney injury, and hospital acquired infection.

In the present study, shorter duration of ICU stay (below 3 days) was strongly associated with ICU mortality. This is in congruent with previous reports from Ethiopia and Nigeria (3,4,7,8). However, these findings contradict studies from the developed region (9). According to a study done by Kevin et al. 2006, patients with prolonged ICU admissions were nearly twice as likely to die as patients with shorter ICU admissions (25% vs 13% p=0.0001) (9). The observed difference could be due to the universal lack of standard pre-hospital and ICU care facilities and trained critical care specialist in the resource limited setting. Furthermore, in the developing countries patients often present late and already complicated. Thus, the cumulative effect of late presentation and poor critical care setting will result in death of patients within few days of ICU admission. Therefore, it's important to upgrade the critical care units with the required equipment, emergency medications, and trained critical care specialist; in order to reduce such high ICU mortality rate.

In this review, nearly quarter of those patients admitted to the ICU required mechanical ventilator support. Furthermore, the proportion of ICU deaths was higher for those patients on respiratory support compared to patients who did not require ventilation support. In multivariate logistic analysis, the need for mechanical ventilation was associated with ICU deaths. These findings were consistent with previous studies (3,4,8,10,11). These results highlight on the need to improve our ICU care service, especially for those who requires respiratory support to reduce the mortality associated with it.

Findings from our study also indicated that, the Presence of aspiration pneumonia, hospital acquired infection, and acute kidney injuries were associated with increased odds of hospital mortality compared to absences of these diagnoses. These findings are similar to previous local and global studies (12–16). These could possibly be due to inadequate medical supplies, shortage of skilled manpower and poor triage system and furthermore more severely ill patients with comorbidities tend to be admitted to ICU, further affecting the outcome (16,17). The limitations of the present study include the retrospective nature of the study, small sample size, and lack of control group for comparison.

The present study shows high prevalence of ICU mortality among adults admitted to the general intensive care unit of Hawassa University Comprehensive Specialized Hospital. Cardiovascular disorders were the leading cause of admissions and death. Furthermore, the presence of acute kidney injury, aspiration pneumonia, and hospital acquired infection and need for mechanical ventilation was significantly associated with increased ICU mortality. Based on our study results, we recommend giving special emphasis to patients in ICU who developed these predictors of mortality, in order to reduce the high ICU mortality observed in the present survey. In addition, we recommend conducting future prospective controlled study to consolidate our findings.

## References

[1] Asrat Agalu, Woldie Mirkuzie, Ayele Yemane, Bedada Worku (2014). Reasons for admission and mortalities following admissions in the intensive care unit of a specialized hospital, in Ethiopia. International Journal of Medicine and Medical Sciences.

[2] Tesema HG, Lema GF, Mesfin N, Fentie DY, Arefayne NR (2021). Patterns of Admission and Clinical Outcomes Among Patients Admitted to Medical Intensive Care Unit of a Teaching and Referral Hospital, Northwest Ethiopia. Glob Adv Health Med.

[3] Onyekwulu FA, Anya SU (2015). Pattern of admission and outcome of patients admitted into the Intensive Care Unit of University of Nigeria Teaching Hospital Enugu: A 5-year review. Nigerian journal of clinical practice.

[4] Gidey K, Hailu A, Bayray A (2018). Pattern and outcome of medical intensive care unit admissions to Ayder comprehensive specialized hospital in Tigray, Ethiopia. Ethiopian Medical Journal.

[5] Ababa A (2014). One year retrospective review of disease patterns and clinical outcomes of patients admitted in intensive care units of Armed Force General Teaching Hospital in Department : Emergency Medicine.

[6] Agalu A, Woldie M, Ayele Y, Bedada W (2014). Reasons for admission and mortalities following admissions in the intensive care unit of a specialized hospital, in Ethiopia. International Journal of Medicine and Medical Sciences.

[7] Woyessa AH, Dibaba BY, Hirko GF, Palanichamy T (2019). Spectrum, pattern, and clinical outcomes of adult emergency department admissions in selected hospitals of Western Ethiopia: a hospital-based prospective study. Emergency medicine international.

[8] Laupland KB, Kirkpatrick AW, Kortbeek JB, Zuege DJ (2006). Long-term mortality outcome associated with prolonged admission to the ICU. The American College of Chest Physicians.

[9] Kedir S, Berhane A, Bayisa T, Wuletaw T (2017). Admission patterns and outcomes in the medical intensive care unit of St. Paul's hospital millennium medical college, Addis Ababa, Ethiopia. Ethiopian medical journal.

[10] Khan U, Menezes CN, Govind N (2021). Patterns and outcomes of admissions to the medical acute care unit of a tertiary teaching hospital in South Africa. African J Emerg Med.

[11] Ibrahim A, Ahmed MM, Kedir S, Bekele D (2016). Clinical profile and outcome of patients with acute kidney injury requiring dialysis – an experience from a haemodialysis unit in a developing country. BMC Nephrol.

[12] Skinner DL, Hardcastle TC, Rodseth RN, Muckart DJJ (2014). The incidence and outcomes of acute kidney injury amongst patients admitted to a level I trauma unit. Injury.

[13] Mandelbaum T, Scott D J, Lee J, Mark R G, Malhotra A, Waikar S S, Howell M D, Talmor D (2011). Outcome of critically ill patients with acute kidney injury using the Acute Kidney Injury Network criteria. Critical care medicine.

[14] Hashemian SM, Jamaati H, Bidgoli BF, Farrokhi FR, Malekmohammad M, Roozdar S, Mohajerani SA, Bagheri A, Radmnand G, Hatami B, Chitsazan M (2016). Outcome of acute kidney injury in critical care unit, based on AKI network. Tanaffos.

[15] Vincent JL, Rello J, Marshall J, Silva E, Anzueto A, Martin CD, Moreno R, Lipman J, Gomersall C, Sakr Y, Reinhart K, EPIC II Group of Investigators (2009). International study of the prevalence and outcomes of infection in intensive care units. JAMA.

[16] Ylipalosaari P, Ala-Kokko TI, Laurila J, Ohtonen P, Syrjälä H (2006). Intensive care acquired infection is an independent risk factor for hospital mortality: a prospective cohort study. Critical Care.

